# Induction of Osmoregulation and Modulation of Salt Stress in *Acacia gerrardii* Benth. by Arbuscular Mycorrhizal Fungi and *Bacillus subtilis* (BERA 71)

**DOI:** 10.1155/2016/6294098

**Published:** 2016-08-15

**Authors:** Abeer Hashem, E. F. Abd_Allah, A. A. Alqarawi, A. A. Al-Huqail, M. A. Shah

**Affiliations:** ^1^Department of Botany and Microbiology, Faculty of Science, King Saud University, Riyadh 11451, Saudi Arabia; ^2^Mycology & Plant Disease Survey Department, Plant Pathology Research Institute, ARC, Giza 12511, Egypt; ^3^Department of Plant Production, Faculty of Food & Agricultural Sciences, P.O. Box 2460, Riyadh 11451, Saudi Arabia; ^4^Seed Pathology Department, Plant Pathology Research Institute, ARC, Giza 12511, Egypt; ^5^Department of Botany, University of Kashmir, Srinagar, Jammu and Kashmir 190001, India

## Abstract

The role of soil microbiota in plant stress management, though speculated a lot, is still far from being completely understood. We conducted a greenhouse experiment to examine synergistic impact of plant growth promoting rhizobacterium,* Bacillus subtilis* (BERA 71), and arbuscular mycorrhizal fungi (AMF) (*Claroideoglomus etunicatum*;* Rhizophagus intraradices*; and* Funneliformis mosseae*) to induce acquired systemic resistance in Talh tree (*Acacia gerrardii* Benth.) against adverse impact of salt stress. Compared to the control, the BERA 71 treatment significantly enhanced root colonization intensity by AMF, in both presence and absence of salt. We also found positive synergistic interaction between* B*.* subtilis* and AMF* vis-a-vis* improvement in the nutritional value in terms of increase in total lipids, phenols, and fiber content. The AMF and BERA 71 inoculated plants showed increased content of osmoprotectants such as glycine, betaine, and proline, though lipid peroxidation was reduced probably as a mechanism of salt tolerance. Furthermore, the application of bioinoculants to Talh tree turned out to be potentially beneficial in ameliorating the deleterious impact of salinity on plant metabolism, probably by modulating the osmoregulatory system (glycine betaine, proline, and phenols) and antioxidant enzymes system (SOD, CAT, POD, GR, APX, DHAR, MDAHR, and GSNOR).

## 1. Introduction

Salinity is a common environmental stress that has adverse impacts on crop growth and development [1, 2], reduces yield of crops, and disrupts the local ecological balance [3]. Plants have been shown to combat the salinity stress through the enzymatic and nonenzymatic antioxidant defense systems that allow the scavenging of reactive oxygen species (ROS) for dynamic equilibrium [4]. Nonenzymatic antioxidant defense systems in plants include glutathione (GSH), phenolic compounds, and alkaloids [5]. Furthermore, the antioxidants act as a substrate for the antioxidant enzymes in the removal of ROS and hence play a direct role in the elimination of ROS [6]. On the other hand, the enzymatic defense system scavenges ROS and repairs the damage caused by ROS [7, 8]. The main antioxidant enzymes are superoxide dismutase, guaiacol peroxidase, glutathione peroxidase, catalase, glutathione reductase, ascorbate peroxidase, monodehydroascorbate reductase, and dehydroascorbate reductase [9].

Recent studies have shown that the colonization of plant roots with PGPR and AMF increases plants tolerance to salinity [10] through induction of osmoregulation and modulation of the impact of salt stress [2, 11, 12]. Plants inoculated with AMF show lower lipid peroxidation and enhanced antioxidant enzyme activities in common bean [13] as systemic resistance tool against salt stress. Similarly, PGPRs have been reported to alleviate salt stress in host plants through multiple mechanisms, including the rapid upregulation of conserved salt stress responsive signaling pathways [14].

Encouraging the vegetation to grow in hot desert conditions is a challenge which becomes even more important when the target plants are economically significant. The genus* Acacia* generally, and* Acacia gerrardii* particularly, is considered one of the most important tree groups in Saudi Arabia because the species is a good source of gums and tannins, besides being used as wood and forage [15]. However, this plant species has been found to be negatively influenced by salt stress conditions which tells upon its population size and distribution status [16]. Hence, we aimed to elucidate the salt stress defense and tolerance mechanisms of* A*.* gerrardii* by mutualistic interaction with endophytic* B. subtilis* and AMF.

## 2. Materials and Methods

### 2.1. Plants, Soil, and Endophytic Microorganisms

Seeds of Talh tree (*Acacia gerrardii* Benth.) were provided by a nursery in Alghat city, Riyadh, that produces tree seedlings. The soil used was loamy sand type with the following properties (%): sand (87.6); clay (7.2); silt (5.2); organic carbon (0.12); total nitrogen (0.005); pH 7.5. The surface of seeds was sterilized with concentrated sulfuric acid followed by 70% (v/v) ethanol for 3 min and rinsed several times with sterile water. The sand was washed with 1.0 N H_2_SO_4_ for one hour, followed by 1.0 N Ca carbonate, and was washed using distilled water. The surface-sterilized seeds were sown in acid-washed sterile sand and kept in a plant growth chamber under the trap culture protocol for one month.

The endophytic bacterium (*Bacillus subtilis* BERA 71) isolated from roots of Talh trees was collected from the top 20 cm of soil in a natural meadow at Khuraim in Riyadh, Saudi Arabia. The 16S rDNA sequence of* Bacillus subtilis* BERA 71 was submitted to the GenBank nucleotide sequence database and the accession number is KX090253 (http://www.ncbi.nlm.nih.gov/nuccore/KX090253?report=GenBank).* Bacillus subtilis* BERA 71 (Bs) was formulated as described in detail by Hashem et al. [17]. The lyophilized bacterial growth was mixed with talc powder (1.0% carboxy methyl cellulose as an adhesive agent) to give a final concentration of 3.6 × 10^9^ cfu (colony forming units) of Bs/gram of formulated material. The bacterial formulated material was suspended in phosphate buffered saline solution and diluted to a cell density of 10^8^ cells/mL.

The AMF (*Claroideoglomus etunicatum* [syn.* Glomus etunicatum*];* Rhizophagus intraradices* [syn.* Glomus intraradices*]; and* Funneliformis mosseae* [syn.* Glomus mosseae*]) used in the present study were extracted from soil samples surrounding the roots of* A*.* gerrardii* by wet sieving, decanting, and sucrose density gradient centrifugation as reported by Daniels and Skipper [18] and modified by Utobo et al. [19]. AMF species were identified based on subcellular structures (spore color, shape, surface ornamentation, spore contents, and wall structures) of asexual spores provided by the international collection of vesicular and arbuscular mycorrhizal fungi [20] and other descriptive protocols [21–23]. The trap culture protocol suggested by Stutz and Morton [24] was used in the current study to propagate the selected mycorrhizal isolates. The trap culture was used as mycorrhizal inoculum and was added to the experimental soil as 25 g of trap soil culture (approx. 100 spores/g trap soil, M = 80%)/pot. Soils not inoculated with mycorrhiza and PGPR served as the control.

### 2.2. Plant Growth Condition

The pot experiment was carried out in the growth chamber of the Plant Production Department, Faculty of Food & Agricultural Sciences, King Saud University, Riyadh, Saudi Arabia. The soil used was loamy sand soil with the following properties (%): sand (87.6); clay (7.2); silt (5.2); organic carbon (0.12); total nitrogen (0.005); pH 7.5. Talh tree seeds were provided by a personal nursery in Alghat city, Riyadh, that produces tree seedlings. The seedlings were transplanted in plastic pots (2 Kg in capacity, one seedling/pot) kept in a plant growth chamber at 25 ± 2°C with an 18 h photoperiod, 750 *μ*mol m^−2^ s^−1^ photosynthetic photon flux density, and 70–75% relative humidity for three months. Half-strength Hoagland's solution was used to irrigate the pots. The experiment was a completely randomized design with five replicates for each treatment: (i) control without microbes, (ii) Bs, (iii) AMF, and (iv) Bs + AMF. Similar treatments with salt were used as references. Salinity was established by adding NaCl to the irrigation solution to obtain a constant concentration of 250 mM. Plants were grown in a greenhouse for 12 weeks after transplantation. At harvest, plants were harvested carefully, and roots were separated from shoots to estimate the intensity of structural colonization. The shoot was dried to constant weight at 100°C to estimate the tools of nutritional value. Fresh leaf samples were used to assess the content of other chemical and biochemical analyses.

### 2.3. Determination of Arbuscular Mycorrhizal Colonization

The fine roots were collected from the lateral root system of each treatment and were fixed in FAA solution, until their further processing. Roots were stained with trypan blue in lactophenol [25] and assessed for mycorrhizal infection. More pigmented roots, after clearing, were bleached in alkaline hydrogen peroxide (0.5% NH_4_OH and 0.5% H_2_O_2_ v/v in water) to remove any phenolic compounds [26] before acidification. For the assessment of mycorrhizal colonization, stained root segments were mounted on glass slides with lactophenol and observed under digital microscope (Model DP-72, Olympus) at 20x magnification. A minimum of 50 segments for each replicate sample were observed for the assessment of structural colonization of AM fungi associated with roots. Twenty or more segments (depending on the slide size) were mounted on each slide and examined under microscope. Presence of mycelium, vesicles, and arbuscules was recorded and analyzed for determining the structural colonization. The intensity of colonization was measured as poor (P), moderate (M), and abundant (A) type of colonization with each of the individual structures as described by Kormanik and McGraw [26] and McGonigle et al. [27]. Intensity of AMF colonization (mycelium, vesicles, and arbuscules) in Talh roots was calculated according to the following formula:

(1) %  Colonization=Total number of AM positive segmentsTotal number of segments studied×100.

### 2.4. Estimation of Nutritional Value

Fine powdered shoot samples were used for estimation of total nitrogen, ash content, and total lipids according to the protocol of Association of Official Agricultural Chemists [28]. Neutral detergent fiber (NDF), acid detergent fiber (ADF), and acid detergent lignin (ADL) were determined according to the method of Van Soest and Robertson [29]. The determination of condensed tannins (HCl-Butanol) was carried out spectrophotometrically (550 nm) according to the method described in detail by Makkar [30]. Spectrophotometer (T80 UV/VIS Spectrometer, PG Instruments Ltd., USA) was used for estimation of color intensity.

### 2.5. Estimation of Lipid Peroxidation (Malondialdehyde, MDA)

0.1 g leaves were homogenized with 1 mL of 10% trichloroacetic acid and centrifuged at 10000 g for 10 minutes. 1 mL supernatant was mixed with 20% trichloroacetic acid containing 0.25% thiobarbituric acid and was heated at 95°C for 30 min, quickly cooled in an ice bath, and then centrifuged again at 10,000 ×g for 10 min in a refrigerated centrifuge at 4°C. The absorbance of the supernatant was read at 532 and 600 nm [31]. Calculations were done using an extinction coefficient of 155 mM cm^−1^.

### 2.6. Estimation of Hydrogen Peroxide (H_2_O_2_) Content

H_2_O_2_ content of leaf samples was calorimetrically measured as described by Mukherjee and Choudhuri [32]. Leaf samples were extracted with cold acetone. 200 *μ*L aliquot was mixed with 0.04 mL of 0.1% TiO_2_ and 0.2 mL NH_4_OH (20%). The pellet was decollated with acetone and resuspended in 0.8 mL H_2_SO_4_. The mixture was then centrifuged at 6,000 ×g for 15 min and supernatant was read at 415 nm.

### 2.7. Estimation of Total Soluble Phenolics

Total phenolics were extracted with 80% (v/v) acetone and estimated using Folin and Ciocalteu's phenol reagent following Slinkard and Singleton [33]. Optical density of the mixture was read at 750 nm. Computation was done from standard curve of pyrogallol. Total soluble phenolics were expressed as mg/g on fresh weight basis.

### 2.8. Glycine Betaine (GB) Content

Dry powdered leaf material (0.5 g) was shaken in 10 mL toluene (0.5%) and was left overnight at 4°C. After centrifugation, one mL of the filtrate was added to 1 mL of sulfuric acid (2 N) and from this 0.5 mL was taken in a test tube and potassium tri-iodide (200 *μ*L) solution was added. The contents were cooled in a chiller and 2.8 mL of ice cooled deionized H_2_O was added followed by addition of 5 mL of 1-2 dichloroethane. Thereafter the absorbance of the organic layer (lower layer) was recorded spectrophotometrically (T80 UV/VIS Spectrometer, PG Instruments Ltd., USA) at 365 nm [34]. Concentrations of GB were calculated from a standard curve of GB.

### 2.9. Estimation of Proline

Free proline was estimated following the method of Bates et al. [35]. 0.5 gm leaf was extracted in sulfosalicylic acid (3%) followed by centrifugation at 3000 g for 30 minutes. 2.0 mL supernatant was mixed with equal volume of acid ninhydrin solution [1.25 g ninhydrin dissolved in 30 mL glacial acetic acid and 20 mL of 6 M phosphoric acid] and glacial acetic acid. The samples were then incubated at 100°C for 10 min and reaction was terminated by keeping the tubes in ice container. After cooling, proline was separated with 4 mL toluene and optical density was measured at 520 nm.

### 2.10. Extraction and Estimation of Enzymatic Antioxidants

The method of Malik and Singh [36], described in detail by Alqarawi et al. [37], was used for extraction of antioxidant enzymes from fresh plant leaves. Protein in the enzyme extract was estimated according to Lowry et al. [38]. The method of Giannopolitis and Ries [39] was used to estimate superoxide dismutase (SOD, EC 1.15.1.1) activity measuring its ability to inhibit the photochemical reduction of nitroblue tetrazolium. The reaction mixture was incubated under light (20 W fluorescent lamps) for 20 min. The absorbance was measured spectrophotometrically at 540 nm. The results were expressed as EU mg^−1^ protein. The method of Chance and Maehly [40] was used for estimation of catalase (CAT, EC 1.11.1.6) activity following the decrease in optical density of the reaction mixture every 20 seconds for 2 min spectrophotometrically at 240 nm and the activity was expressed as EU mg^−1^ protein. Peroxidase (POD, EC 1.11.1.7) was estimated according to the method described by Kar and Mishra [41]. The assay mixture comprised phosphate buffer (125 *μ*M, pH 6.8), pyrogallol (50 *μ*M), H_2_SO_4_ (50 *μ*M), and 50 *μ*L of enzyme extract. The reaction mixture was incubated at 25°C for 5 min; thereafter 0.5 mL of 5% (v/v) H_2_SO_4_ was added to stop the enzyme reaction. The end product (purpurogallin) was determined spectrophotometrically at 420 nm and activity was expressed as EU mg^−1^ protein. The method of Nakano and Asada [42] was followed to estimate the activity of ascorbate peroxidase (APX, EC 1.11.1.11) by monitoring change in absorbance spectrophotometrically at 290 nm and activity was expressed as EU mg^−1^ protein. The activity of glutathione reductase (GR, EC 1.6.4.2) was assayed as described by Carlberg and Mannervik [43] spectrophotometrically at 340 nm. The activity of GR was calculated using the extinction coefficient of 0.12 mM NADPH of 6.2 mM^−1^ cm^−1^ and expressed as EU mg^−1^ protein as described by Ahmad et al. [44]. For extraction nitrosoglutathione reductase (GSNOR, EC 1.1.1.284) enzyme, fresh leaves were homogenized in an assay mixture containing Tris-HCl (20 mM, pH 8.0), NADH (0.2 mM), and EDTA (0.5 mM) at 4°C [45]. The homogenate was centrifuged for 10 min at 3000 g. The supernatant was used as enzyme source. The method of Bai et al. [46], described in detail in our previous work by Cheng et al. [45], was used to estimate the activity of GSNOR spectrophotometrically at 340 nm at 25°C by monitoring the oxidation of NADH nm. The reaction was initiated by adding S-nitrosoglutathione (Calbiochem, San Diego, CA, USA) to the supernatants at a final concentration of 400 mM. The enzyme activity was expressed as nanomoles NADH consumed per minute per milligram of protein as described by Cheng et al. [45]. The method of Nakano and Asada [42] was used for determination of dehydroascorbate reductase (DHAR, EC: 1.8.5.1) activity and the absorbance was read at 265 nm for 1 min spectrophotometrically using extinction coefficient of 14 mM^−1^ cm^−1^ as described by Ahmad et al. [44]. The method of Miyake and Asada [47] was employed for the estimation of monodehydroascorbate reductase (MDHAR, EC 1.6.5.4). MDAR activity was expressed as *μ*mol NADPH oxidized/(EU mg^−1^ protein) as described by Cheng et al. [45].

### 2.11. Statistical Analyses

Duncan's multiple range test was performed using one-way analysis of variance (ANOVA) for a completely randomized design by SPSS-21 software and significant differences in means were determined by the least significant differences (LSD) (*p* = 0.05) test. *p* ≤ 0.05 was considered as significant [48]. Data presented are the means ± SE (*n* = 5).

## 3. Results

### 3.1. Host Plant-Soil Biota Interactions

**Figure 1:**
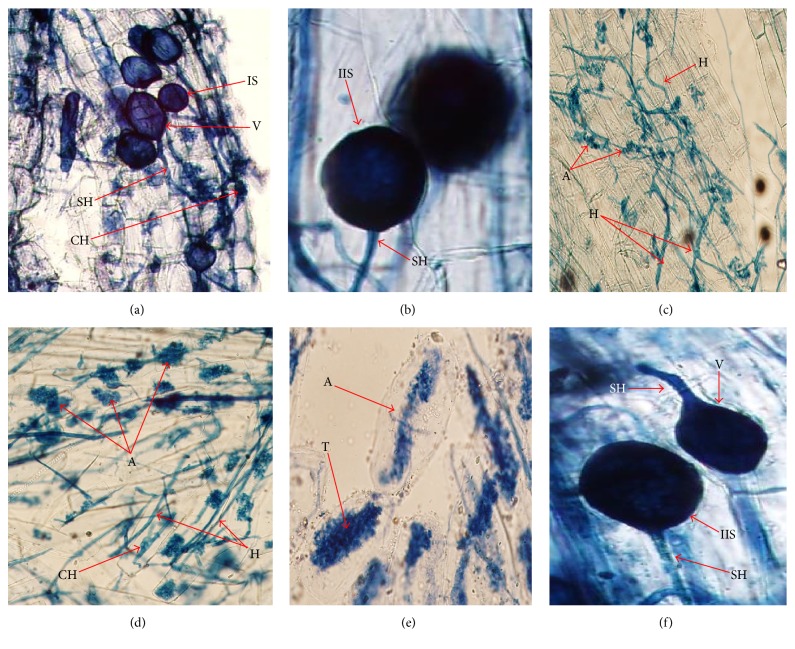
Illustration of different arbuscular mycorrhizal fungi colonization in root of* Acacia gerrardii*. (a) Interadical spores (IS), coiled hyphae (CH), and vesicles (V) [20x]. (b) Intact interadical spores (IIS) and subtending hyphae (SH) [40x]. (c) Arbuscules (A) and hyphae (H) [10x]. (d) Arbuscules (A), hyphae (H), and coiled hyphae (CH) [10x]. (e) Arbuscules (A) and trunk (T) [40x]. (f) Vesicle (V), intact interadical spores (IIS), and subtending hyphae (SH) [40x].

AMF colonized roots of* A. gerrardii* with good intensity revealed by the presence of mycelia, vesicles, and arbuscules (Figures 1(a)–1(f)). Generally, the incidence of root length colonization (mycelia and arbuscules) by AMF was found to be relatively poor (P) with percent colonization of 51.2 and 63.6, respectively. However, the incidence of dormant status (vesicles) was moderate (M) to the tune of 50.4%. The application of Bs stimulated the intensity AMF colonization in the order of 120.7%, 48.5%, and 383.7% for mycelia, vesicles, and arbuscules, respectively, as compared to control. The intensity of abundant and moderate colorization of AMF as mycelium, vesicles, and arbuscules was negatively affected by salinity but inoculation with Bs significantly reduced the adverse impacts of salinity on the AMF structures (mycelium and arbuscules) in root of* A*.* gerrardii* (Table 1). The application of Bs significantly stimulated the structural colorization of AMF under saline and nonsaline conditions in terms of visible increase in the incidence of mycelia, vesicles, and arbuscules. The inoculated roots, however, showed significant decrease in mycelium, vesicles, and arbuscules, respectively, both in saline (34.4%, 52.9%, and 13.6%) and in nonsaline conditions (81.6%, 42.8%, and 64.77%), compared to the respective control (Table 1).

### 3.2. Nutritive Value

In absence of salt stress (control), inoculation of* A*.* gerrardii* with AMF and Bs in combination showed the highest impact on nutritive value compared to their individual treatments. Plants treated with salt suffered a loss in nutritive value as indicated by an increase in ash, tannin, and lignin content by 40.1%, 261.8%, and 72.6%, respectively, though total lipids were reduced by 65.4% compared to the control (Table 2). Also, neutral detergent fiber (NDF) decreased by 46.1% and acid detergent fiber (ADF) increased by 29.8% in salt treated plants (Table 2). The loss of nutritive value was lower in presence of AMF and Bs under salt stress compared to the corresponding controls. It was observed that the combination between AMF and Bs was more positively effective to improve the nutritive value in absence (control) of salt stress. However, the salinity caused slight inhibition in the positive impact of AMF and Bs.

### 3.3. Hydrogen Peroxide (H_2_O_2_) and Malonaldehyde (MDA)

**Figure 2:**
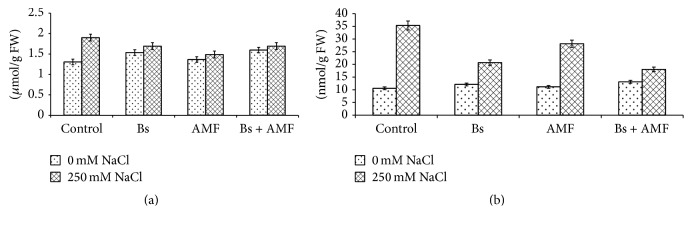
Effect of salinity stress (250 mM NaCl) on (a) hydrogen peroxide [H_2_O_2_] (LSD at 0.05 : 0.025; coefficient of variance: 1.867) and (b) malonaldehyde [MDA] (LSD at 0.05 : 0.567; coefficient of variance: 3.518) in* Acacia gerrardii* with and without AMF and* Bacillus subtilis *(Bs).

As shown in Figures 2(a) and 2(b), H_2_O_2_ Figure 2(a) and MDA Figure 2(b) were negatively affected by salinity and significant increase by 45.6% and 234.1% was, respectively, observed as compared to the nonsaline control. But inoculation with AMF and Bs significantly reduced the adverse effects of salinity on both H_2_O_2_ and MDA.

It is evident that inoculation with AMF or Bs only (in absence of salt stress) caused slight increase in H_2_O_2_ and MDA, though inoculation with AMF and Bs together caused more increase compared to individual inoculation and nontreated control plants.

### 3.4. Glycine Betaine and Proline

**Figure 3:**
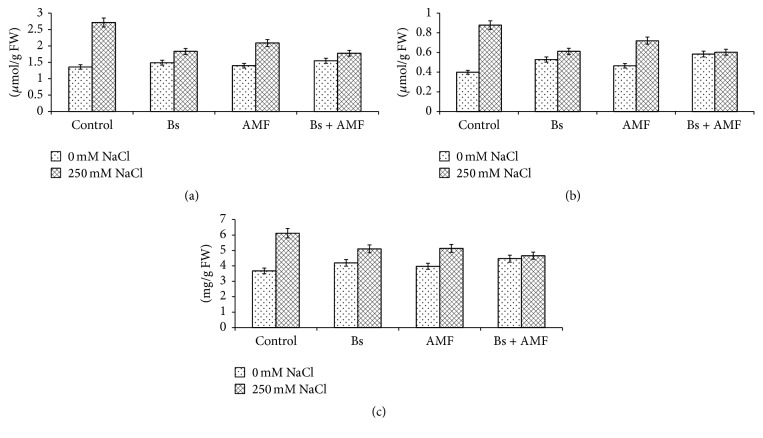
Effect of salinity stress (250 mM NaCl) on (a) glycine betaine (LSD at 0.05 : 0.162; coefficient of variance: 10.567), (b) proline (LSD at 0.05 : 0.026; coefficient of variance: 5.023), and (c) total phenol (LSD at 0.05 : 0.109; coefficient of variance: 2.705) in* Acacia gerrardii* with and without AMF and* Bacillus subtilis *(Bs).

Effect of salinity, AMF, and Bs on glycine betaine and proline is depicted in Figures 3(a) and 3(b). Glycine, betaine, and proline increased by 100% and 125.1% in salinity stressed plants (Figures 3(a) and 3(b)). However, in salt stressed AMF inoculated (AMF + 250 mM NaCl) plants showed an increase of 54.07% and 82.05% while as in salt stressed Bs inoculated (250 mM NaCl + Bs) treatment showed 38.4% and 56.7% increase in glycine betaine and proline, respectively (Figures 2(a) and 2(b)). In salt stressed conditions inoculation with AMF and Bs (250 mM NaCl + AMF + Bs) showed only 31.1% and 54.5% increase in H_2_O_2_ and MDA production (Figures 2(a) and 2(b)). Individually inoculation of AMF induced 2.9% and 16.55% increase while Bs induced 9.6% and 33.1% increase in glycine betaine and proline, respectively (Figures 3(a) and 3(b)).

### 3.5. Total Phenol

Salinity stress increased phenol content by 69.6% while AMF and Bs inoculated plants showed an increase of 8.3% and 13.8%, respectively (Figure 3(c)). AMF and Bs inoculated salt stressed (AMF + Bs + 250 mM NaCl) treatments showed an increase of the order of 27.7%.

### 3.6. Antioxidant Enzyme Activity

**Figure 4:**
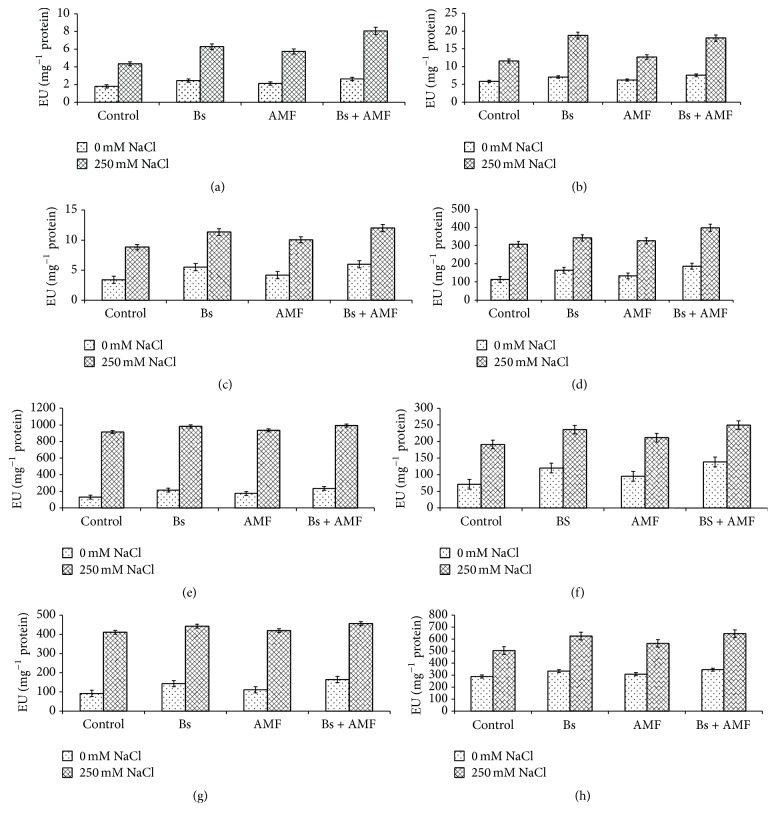
Effect of salinity stress (250 mM NaCl) on (a) superoxide dismutase [SOD, EC 1.15.1.1] (LSD at 0.05 : 0.069; coefficient of variance: 1.910); (b) catalase [CAT, EC 1.11.1.6] (LSD at 0.05 : 0.173; coefficient of variance: 1.830); (c) peroxidase [POD, EC 1.11.1.7] (LSD at 0.05 : 0.238; coefficient of variance: 3.597); (d) glutathione reductase [GR, EC 1.6.4.2 (nmol/min/g FW)] (LSD at 0.05 : 4.056; coefficient of variance: 1.903); (e) ascorbate peroxidase [APX, EC 1.11.1.11, nmol/min/g FW] (LSD at 0.05 : 6.907; coefficient of variance: 1.394); (f) dehydroascorbate reductase (DHAR, EC: 1.8.5.1, nmol/g FW) (LSD at 0.05 : 2.492; coefficient of variance: 1.751); (g) monodehydroascorbate reductase [MDHAR, EC 1.6.5.4, nmol/min/g FW] (LSD at 0.05 : 4.374; coefficient of variance: 1.805); and (h) nitrosoglutathione reductase [GSNOR, EC 1.1.1.284, nmol/g FW] (LSD at 0.05 : 4.335; coefficient of variance: 1.108) in* Acacia gerrardii* with and without AMF and* Bacillus subtilis *(Bs).

Results depicting the effect of salinity, AMF, and Bs alone, as well as in combination, on the activities of antioxidant enzymes are shown in Figures 4(a)–4(g). Activities of the antioxidants studied (SOD, CAT, POD, GR, APX, DHAR, MDAHR, and GSNOR) increased with AMF, Bs, and salinity stress. Relative to control, percent increase in SOD, CAT, POD, GR, APX, DHAR, MDAHR, and GSNOR for AMF inoculated was 23.5%, 6.8%, 24.2%, 17.6%, 33.5%, 50.1%, 20.6%, and 6.8%, respectively. On the other hand, in case of AMF and Bs inoculated plants enhancement was much more pronounced with percent increase of 41.1%, 22.9%, 66.3%, 45.3%, 63.3%, 69.4%, 55.1%, and 16.2% with respect to SOD, CAT, POD, GR, APX, DHAR, MDAHR, and GSNOR, respectively. Salt stressed plants showed an enhancement of 152.9%, 98.2%, 166.6%, 169.5%, 598.3%, 169.1%, 350.5%, and 75.6% in SOD, CAT, POD, GR, APX, DHAR, MDAHR, and GSNOR activity, respectively (Figures 4(a)–4(g)). However, inoculation of AMF and Bs to salt stressed plants (250 mM NaCl + AMF + Bs) resulted in marked enhancement in activities of antioxidant enzymes studied and the percent increase being 374.7%, 210.3%, 263.6%, 251.8%, 656.4%, 178.1%, 401.1%, and 124.7% for SOD, CAT, POD, GR, APX, DHAR, and MDAHR, respectively, which is more as compared to the control as well as salt stressed plants and the AMF or Bs inoculated plants (Figures 4(a)–4(g)).

## 4. Discussion

Inoculation by AMF and Bs increased the tissue water content and the nutrient uptake and caused hormonal balance in the treated plants. This resulted in optimal activity of metabolic processes to meet the growing needs of host plants for coping with the stress as reflected by other studies [4, 49]. Our results of reduction in percent AMF colonization and related attributes such as spore density due to salinity support the findings of Aroca et al. [2] and Alqarawi et al. [37]. The synergistic interactions of AMF and PGPR were evidenced by the fact that Bs supported significant increase in incidence of AMF colonization either in saline or in nonsaline conditions. The production of plant growth hormones by Bs plays direct and indirect role in promotion of mycorrhizal colonization. On the other hand, Bs may have more essential role in management of ROS [50] which in turn tends to decrease the affinity between AMF and host plant [14].

Salt stressed plants showed higher production of MDA, a product of lipid peroxidation, reflecting thereby the reduced membrane stability and impaired membrane functioning. By and large similar results have been obtained by Ahmad et al. [44] for* Brassica juncea*. In* Phaseolus vulgaris* [13] and* Ephedra alata* [51] it has been demonstrated that colonization by AMF reduces the peroxidation of membrane lipids, thereby providing stability to membranes against salt stress. Improved stability to lipids from the oxidative degradation of toxic reactive oxygen species due to AMF and PGPR in salt stressed plants is due to the enhancement in the free radical scavenging mechanisms [13, 51]. Tuna et al. [52] have confirmed that higher salinity enhances the membrane leakage through increased peroxidation of lipids. Harsh environmental conditions induce excessive production as well as accumulation of reactive oxygen species (ROS) and ultimately triggering the peroxidation of unsaturated membrane lipids thereby resulting in the loss of membrane integrity which ultimately leads to leakage and desiccation [44, 53]. Recently, Ahmad et al. [44] and Abd Allah et al. [13] observed increased production of free radicals like H_2_O_2_ in salt stressed plants and subsequent reversal of their damaging effects by AMF and PGPR to some extent. Improvement in membrane stability reduced MDA content in AMF inoculated plants may be due to the substantial increase in antioxidant activities and phosphate metabolism [13, 37]. In addition to reducing the cell membrane index and cell caspases, PGPR inoculation provides salt tolerance through downregulating the protease activity and programmed cell death resulting in enhanced cell viability [54].

Stressed environmental conditions trigger the accumulation of proline, glycine betaine, and other compatible osmolytes in plants. Accumulation of organic solutes including proline, sugars, and glycine betaine is one of the important tolerance strategies adapted by plants during stressful conditions [4]. In the present study, increase in content of proline and glycine betaine under salinity stress is in corroboration with Ahmad et al. [9, 44]. In* Ephedra aphylla* [37] and* Sesbania sesban* [13] accumulation of proline leads to salinity stress amelioration through better extraction of water from the soil solution by its active role in osmotic adjustment. They also reported that, under both normal and salt stressed conditions, inoculation of AMF induced enhanced synthesis of proline which helps in stress adaptation. Under salt stress, accumulation of proline results from the upregulation of the enzymes involved in proline synthesis which is concomitant with the downregulation of the catabolizing enzymes [55]. Proline accumulation can also result from the reduction in its incorporation within the proteins during protein synthesis or degradation of the existing proteins [56]. Among the important roles of proline and glycine betaine is the maintenance of tissue water balance of plants so that the stress induced ravage gets insulated, in addition to the antioxidant potential to mediate scavenging of the toxic radicals. Besides, optimal concentration of proline and glycine betaine positively influences the protein turnover besides being implicated in the regulation of several stress protective proteins [4, 55]. Under normal as well as salt stressed conditions AMF and PGPR inoculated plants showed enhanced proline accumulation as compared to control and similar results have been reported by Ghorbanpour et al. [57] in* Hyoscyamus niger*. In* Triticum aestivum* introgression of glycine betaine synthesizing gene increases tolerance to multiple stresses like drought, salinity, and cold through the maintained membrane integrity, activity of enzymes, photosynthetic efficiency, and scavenging of ROS [4, 45, 50, 58]. Glycine betaine also stabilizes the constituent proteins of PSII photosynthetic complex and also helps to maintain the order of membranes at extreme temperatures and salinity levels [4].

Accumulation of higher content of phenolic compounds such as lignins, tannins, and fibers is another important strategy for avoiding the stress induced changes. Tannins and phenols are the group of secondary metabolites implicated in plant protection and have been recognized for their antioxidant property. They are involved in eliciting the proper response in plants during biotic and abiotic factors [4, 11, 58]. Improved phenol and tannin content support better growth and also mediate the radical scavenging. In our target plant species inoculation with AMF and Bs enhanced accumulation of phenols and tannin as reflected in enhanced membrane stability in such plants.

Among the antioxidants, SOD has the distinction of scavenging the superoxide radicals. In salt stressed* Acacia gerrardii* increase in activity of SOD was observed which supports the findings of Rasool et al. [59] for* Cicer arietinum *and Alqarawi et al. [37] for* Ephedra aphylla*. Enhanced activity of SOD causes quick conversion of superoxide into H_2_O_2_ through Haber-Weiss reaction and thereby reduces the chances of hydroxyl (OH^−^) radical formation. Efficient removal of free radicals facilitates normal membrane functioning. Higher POD activity in salinity stressed plants corroborates with the results of Abd Allah et al. [13]. Enhanced expression of the POD enzymes results in the increased production of lignins and the associated protective compounds like quinones which ultimately work for reducing the damage induced by oxidative stress. Efficient detoxifications of ROS help plants in maintenance of growth and the physiological activity of plant [4, 12, 13]. Increase in the activity of antioxidant enzymes (SOD and POD) as a result of AMF colonization coincides with the results of Abd Allah et al. [13] for* Phaseolus vulgaris* and Alqarawi et al. [37] for* Ephedra aphylla*. Catalase (CAT) is another important antioxidant enzyme having indispensable role in stress amelioration. In concurrence with our results, increased activity of CAT in salt stressed plants is the result of several workers [13, 51, 59]. Inoculation of AMF and PGPR further enhanced the activities of antioxidant enzymes thereby strengthening the antioxidant defense system. Greater activity of CAT allays the stress damage by mediating the quick removal of H_2_O_2_ [58]. Enhancement in CAT activity as a result of AMF has been reported in soybean [11, 60] and* Ephedra aphylla* [37].* Acacia gerrardii* plants subjected to salt stress showed increased activities of the enzymes of ascorbate-glutathione cycle (APX, MDHAR, DHAR, and GR) resulting in quick generation of redox buffer components, glutathione, and ascorbate, for scavenging of H_2_O_2_. Our results of increased activities of APX, MDHAR, DHAR, and GR support the findings of Ahmad et al. [44]. Ascorbate-glutathione cycle is an important ROS scavenging pathway that involves sequential redox reactions in which both enzymatic and nonenzymatic components are actively engaged for removal of ROS. In this pathway, ascorbate, glutathione, and NADPH serve as electron donors for the efficient activity of enzymatic components, APX, MDHAR, DHAR, and GR [13]. In our study, activity of APX, MDHAR, DHAR, and GR was further increased by the inoculation of AMF and Bs resulting in amelioration of oxidative stress effects of salinity. In* Ocimum basilicum*, Heidari et al. [61] and Heidari and Golpayegani [62] have also demonstrated the upregulation of antioxidant enzyme activities due to inoculation of different PGPR species. Upregulation of antioxidant system and scavenging of ROS go hand in hand and are often correlated with stress tolerance [13, 44, 51]. AMF inoculation induced a significant increment in the antioxidant enzyme activities under normal as well as salt stressed condition. These results are in confirmation with the results of Alqarawi et al. [51] and Sirajuddin et al. [63]. Plants with increased glutathione reductase activity maintain optimal ratio of NADP^+^/NADPH thereby receiving electrons of photosynthetic electron transport chain by molecular oxygen and reducing the formation of superoxide radical [4, 11, 31, 53, 58].

## 5. Conclusion

In conclusion, our results indicate that endophytic bacteria and AMF that live entirely within plant tissues in* A. gerrardii* are jointly involved in ameliorating its tolerance to salt stress. Synergistic interaction of* B*.* subtilis* and AMF enhanced activities of antioxidant enzymes thereby preventing ROS induced oxidative damage. Increase in proline and glycine betaine in AMF and* B*.* subtilis* inoculated plants supports their role for growth improvement of* Acacia gerrardii *under salinity. Hence, a microbial consortium based on these species in combination with other beneficial soil microbes can potentially be used for large scale growth of* A. gerrardii *under salt stressed conditions.

## Figures and Tables

**Table 1:** Effect of salinity stress (250 mM NaCl) on intensity of structural colonization (%) of arbuscular mycorrhizal fungi (AMF) as mycelia (M), vesicles (V), and arbuscules (A) in *Acacia  gerrardii* in response to endophytic *B*. *subtilis* (Bs). Data presented are the means ± SE (*n* = 5).

Treatments		Intensity of structural colonization (%)								
		Mycelium			Vesicles			Arbuscules		
		P	M	A	P	M	A	P	M	A
Control	AMF	51.2	27.6	21.2	22.4	50.4	27.2	63.6	29.0	7.4
	AMF **+** Bs	9.4	43.8	46.8	12.8	34.4	52.8	22.4	41.8	35.8

250 mM NaCl	AMF	77.2	16.8	6	71.4	19.4	9.2	80.8	14.4	4.8
	AMF **+** Bs	50.4	34.4	15.2	33.6	41.2	25.2	69.8	19.4	10.8

LSD at 0.05%		14. 51	8.71	7.04	8.09	9.74	5.11	7.23	5.24	2.07

Coefficient of variance		4.229	3.127	2.531	2.065	4.235	3.011	4.512	3.234	2.107

P = poor; M = moderate; A = abundant.

**Table 2:** Effect of salinity stress (250 mM NaCl) on ash content, total lipids, lignin, tannin, and fiber content (neutral detergent fiber (NDF) and acid detergent fiber (ADF)) in *Acacia gerrardii* with and without arbuscular mycorrhizal fungi (AMF) and *Bacillus subtilis* (Bs). Data presented are the means ± SE (*n* = 5).

Treatments		Nutritional value					
		Ash content (%)	Total lipid content (%)	Lignin	Tannin	Fiber content (%)	
						ADF	NDF
Control	Control	45.0	2.75	46.9	1.65	119.7	277.0
	Bs	32.3	3.52	40.6	1.98	145.7	299.4
	AMF	36.6	2.90	52.4	1.75	132.8	282.0
	AMF + Bs	30.6	3.53	49.6	2.26	154.5	310.7

250 mM NaCl	Control	63.1	0.95	81.0	5.97	148.9	149.1
	Bs	50.1	1.93	61.7	3.14	212.9	213.6
	AMF	57.2	1.37	77.5	4.01	192.4	194.2
	AMF + Bs	48.6	2.23	71.6	2.79	221.1	219.4

LSD at 0.05%		1.62	0.08	1.56	0.16	2.38	4.11

Coefficient of variance		4.136	4.311	2.997	6.305	1.659	1.955
